# Oxytocin’s Regulation of Thermogenesis May Be the Link to Prader–Willi Syndrome

**DOI:** 10.3390/cimb45060313

**Published:** 2023-06-06

**Authors:** Claudia Camerino

**Affiliations:** 1 Department of Biomedical Sciences and Human Oncology, Section of Pharmacology, School of Medicine, University of Bari Aldo Moro, P.za G. Cesare 11, 70100 Bari, Italy; ccamerino@libero.it; 2 Department of Physiology and Pharmacology “V. Erspamer”, Sapienza University of Rome, P.le Aldo Moro 5, 00185 Rome, Italy

**Keywords:** Oxytocin, Prader–Willi Syndrome, thermogenesis, muscle contraction, muscular hypotonia, metabolic syndrome

## Abstract

Prader–Willi Syndrome (PWS) is a genetic neurodevelopmental disorder that is caused by either the deletion of the paternal allele of 15q11-q13, maternal uniparental disomy of chromosome 15 or defects in the chromosome 15 imprinting centre and is characterized by cognitive impairment, hyperphagia and low metabolic rate with significant risk of obesity, as well as a variety of other maladaptive behaviours and autistic spectrum disorder (ASD). Many of the features seen in PWS are thought to be due to hypothalamic dysfunction resulting in hormonal abnormalities and impaired social functioning. The preponderance of evidence indicates that the Oxytocin system is dysregulated in PWS individuals and that this neuropeptide pathways may provide promising targets for therapeutic intervention although the process by which this dysregulation occurs in PWS awaits mechanistic investigation. PWS individuals present abnormalities in thermoregulation an impaired detection for temperature change and altered perception of pain indicating an altered autonomic nervous system. Recent studies indicate that Oxytocin is involved in thermoregulation and pain perception. This review will describe the update on PWS and the recent discoveries on Oxytocin regulation of thermogenesis together with the potential link between Oxytocin regulation of thermogenesis and PWS to create a new groundwork for the treatment of this condition.

## 1. Introduction

Prader–Willi Syndrome (PWS) is a rare neurodevelopmental disorder caused by a loss of paternally expressed genes on chromosome 15q11-q13 [1]. PWS newborn individuals are characterized by hypotonia that can lead to difficulties in sucking and require assisted feeding. Later in life, PWS children develop increased interest in food and hyperphagia and severe obesity that can be fatal if not appropriately controlled. The hypothalamic syndrome of PWS individuals manifests with obesity, body temperature instability, multiple endocrine abnormalities such as growth hormone deficiency with short stature, hypogonadism and hypothyroidism and mild cognitive disability, a behavioral phenotype with temper outbursts and often autistic spectrum disorder (ASD) [2,3,4]. Oxytocin (Oxt) is a neuropeptide secreted by the paraventricular (PVN) and supraoptic (SON) nuclei of the hypothalamus and is involved in PWS [4]. Oxt regulates energy metabolism, the lean/fat composition of the body and thermoregulation. Mice homozygous for deletions of Oxt or its receptor (Oxtr) show late-onset obesity, albeit are normophagic [5,6]. Oxt/Oxtr^−/−^ mice are not obese as newborns but become obese later in life because the obesity caused by the lack of Oxt takes time to reach full force [7]. Oxtr^−/−^ mice have a temperature lower than wild-type, and this was the first piece of evidence that Oxt regulates thermogenesis [8,9,10]. The exposure of wild-type mice to cold temperature increases Oxtr mRNA in the hypothalamus, in white adipose tissue (WAT) and brown adipose tissue (BAT) and decreased circulating Oxt following a negative feed-back in brain [11]. Cold stress (CS) also increases Oxt levels in skeletal muscles. This evidence lets us speculate that the normophagic obesity of the Oxt/Oxtr^−/−^ mice was caused by depotentiation of the skeletal muscle that led to fat infiltration and dysfunctional regulation of body temperature rather than by increased food consumption [11]. Conversely, Oxt infusion increases body temperature [12]. Cold exposure increases Oxt potentiating the slow-twitch muscle consistent with the shivering needs of thermogenesis through the regulation of Myosin heavy chain 1 (slow oxidative)/Myosin heavy chain 2 b (fast glycolytic) ratio triggering what we call “the Oxytonic contraction”. In PWS, Oxt signaling is dysfunctional, and PWS individuals present altered blood levels of Oxt. Moreover, post-mortem studies have shown a reduction in the volume of PVN and a strong decrease in the number of Oxtr-expressing neurons in PVN compared to healthy controls [13]. This phenotype of PWS individuals is the striking mirror image of the phenotype of the cold-stressed mice in our model [11], indicating a link between Oxt functional regulation of thermogenesis and dysfunctional Oxt systems in PWS [11]. Recent studies indicate that the thermosensory system is atypical in Autistic Spectrum Disorder (ASD) [14] since in individuals with ASD, a cold stimulus is perceived as hot or burning and an insensitivity to the outside temperature is reported [15,16]. This abnormal sensory perception could be due to an impairment of small-fiber sensory nerves in patients with ASD [17]. PWS individuals present decreased perception of cold temperatures and increased sensitivity to hot temperatures. The perception of pain related to cold and hot is also altered. Thermoregulation problems are a clinical feature of children with PWS and has been suggested to be linked to fewer Oxt neurons in PVN [2]. Indeed there are several cases of high fever similar to septic shock but with no infections or severe hypothermia in children and adolescents with PWS [14,18,19], as well as in Shaaf-Yang Syndrome (SSY), which has several overlapping features with PWS but is caused by pathogenic/truncating variants of MAGEL2 and the diagnosis of ASD is more frequent [20]. Finally, in the mouse model of SSY (Magel2 mouse), a high sensitivity to cold temperature is also reported that can be corrected by intranasal (IN) administration with Oxt agonist [21]. In this article, we will review the clinical features of PWS, the results from the latest clinical trials with Oxt administration and the new knowledge regarding Oxt regulation of thermogenesis applied to PWS.

## 2. An Update on Prader–Willi Syndrome

### 2.1. Phenotype and Features of Prader–Willi Syndrome 

PWS is a rare complex genetic disorder arising from the lack of expression of paternally inherited genes on chromosome 15q11-q13 due to the deletion of the paternally inherited region (delPWS) or maternal uniparental disomy, where both copies are inherited from the mother (mUPD), or to an imprinting defect due to microdeletions or epimutations and paternal chromosomal rearrangements such as translocations [22]. Magel2 is one of the affected genes located on 15q11-q13, and Magel2 mutations have been found in individuals with ASD, intellectual disability and PWS [23]. Necdin and Magel2 are two mouse models with genetic defects similar to PWS, and they show a similar Oxt dysfunction to that seen in PWS individuals [24]. Indeed, Magel2-deficient mice have a phenotype similar to PWS with impaired suckling at birth that can lead to mortality, reduced Oxt expression in the hypothalamus and feeding problems that are rescued by the administration of a single dose of Oxt a few hours after birth [25]. The social impairment is improved by early intervention with Oxt. PWS individuals present several characteristics that can be simplified into four nutritional phases. Nutritional phase 1a includes decreased appetite and poor suck due to severe hypotonia that causes the need for assisted feeding. Nutritional phase 1b, during which the appetite generally increases [26]. Nutritional phase 2a happens around the age of two, during which body weight increases without a change in food consumption. Nutritional phase 2b around the age of 4–5 when there is an increased interest in food, increased anxiety and behavioral issues. Finally, Nutritional phase 3 at age 8 is characterized by worsening behavior and obesity together with other endocrine problems caused by hypothalamic syndrome [27] (Table 1).

PWS individuals have a dysfunctional Oxt system with less Oxt-producing neurons in the PVN and a decreased Oxtr gene function which impairs the satiety response. Specifically, in adults with PWS the number of Oxt-expressing neurons in the hypothalamus was decreased and plasma level of Oxt is reported altered and, in some cases, low in relation to their obesity. However, in children between 5 and 11 years of age, plasmatic Oxt is reported higher than in healthy siblings [28]. This is in disagreement with the anorexigenic effect of Oxt and can be due to the disruption of Oxt responsiveness, like a sort of Oxt resistance or alternatively to an overproduction of the non-biologically active form of Oxt [29]. The increased production of circulating Oxt in PWS might lead to a compensatory decreased expression of Oxtr in the PVN [1] in a positive loop with brain. PWS individuals have lower insulin levels and resistance than body mass index-matched controls, which may suggest a protected glucose metabolism. Indeed, the prevalence of impaired glucose intolerance, type 2 diabetes mellitus and other obesity-associated complications in patients with PWS tends to be lower when compared to that in general obesity which is consistent with a protected glucose metabolism. The causes are still mysterious although certain features of PWS such as abnormalities in thermoregulation, sleep control and altered pain perception indicate an altered autonomic nervous system (ANS) [30]. Oxt and Oxtr^−/−^ mice have lower adrenalin levels than control [5,6], but whether this altered ANS activity is associated with features of Oxt/Oxtr^−/−^ mice needs to be further researched. Indeed, Oxt plays an essential role in glucose metabolism but the lack of research about Oxt and insulin resistance in PWS prevents further analysis. Moreover, the underlying mechanisms by which Oxt influences glucose metabolism in PWS are unknown [30]. However, one of the first fMRI studies on the satiety response in PWS measured brain activation after ingestion of glucose [31]. The authors showed a delayed response to glucose in PWS, meaning that the brain of individuals with PWS display a delayed satiety effect in various brain regions associated with food intake and reward because of the hypothalamic dysfunction [20]. In this case, supplementation with the active formulation of Oxt could be beneficial, even in the face of elevated serum levels of Oxt.

### 2.2. Pharmacological Intervention with Oxytocin in Prader–Willi Syndrome

Pharmacological interventions in which Oxt was given intranasally to PWS patients are focused on reducing mostly symptoms of hyperphagia, reducing BMI and improving anxiety and behavior in general. The activity of Oxt neurons located in the hypothalamus is involved in feeding behavior in animal studies and could also be altered in PWS patients during food intake. PWS individuals have high circulating Oxt, and, given the anorexic effect of Oxt, it seems plausible that diminished Oxt activity could even worsen hyperphagia, delaying the satiety response [20]. Together these findings suggest that there is an abnormality in the Oxt system in PWS and that the administration of Oxt may have some effects on PWS symptoms. Recently, a series of four clinical trials were performed, and the results were not always linear. In the first study [32], a double-blind placebo-control, crossover study in 24 children aged 5–11 with PWS was performed using 5 days IN-Oxt or 5 days of IN placebo spray, followed by 4 weeks with no medication and then patients returned for an extra 5 days of treatment. In this study, all parameters considered as references improved from day 3 to day 6, favoring Oxt over placebo, but this trend never reached a statistical significance from day 6 to 14. There was no evidence of a difference in lab parameters in Oxt over placebo, showing that the treatment is safe and meaning that the negative results reported in previous studies were probably caused by an overdose of the hormone [32]. This can result in the desensitization of the receptors or increased binding of Oxt to the vasopressin receptor, which can create negative behaviors [27]. The second clinical trial studied the effect of three months IN-Oxt twice daily compared to placebo on 26 children aged 3–11 on hyperphagia and behavior, also investigating the difference between boys and girls and the genetic subtypes [33]. The third clinical trial studied the effect on 23 children aged 5–18 of an 8 week double-blind placebo-controlled IN-Oxt randomized trial [34]. In the second study [33], no significant effects of Oxt on social behavior or hyperphagia were found. However, in boys, Oxt administration showed significant positive effects on hyperphagia, social and eating behavior. In girls, no significant beneficial effect was found. Patients with deletion (delPWS) had significant positive effects, but patients with mUPD did not, probably because the deletion subtypes are associated with more maladaptive behavior. Moreover, the ratio between acylated ghrelin (AG) and unacylated ghrelin (UAG) after Oxt is lower than placebo, showing a decreased orexigenic drive in Oxt-treated patients [33]. The gender differences can be caused by the differences in the Oxt system in boys and girls and also in muscle lean/fat ratio. In the third study [34], the placebo was associated with a modest improvement in hyperphagia and repetitive behavior in children with PWS, whereas IN-Oxt was not associated with any improvement. Moreover, an increase in salivary Oxt at week 4 was positively correlated with an increase in hyperphagia drive but negatively correlated with ritualistic behavior. Low levels of Oxtr in PWS combined with elevated levels of plasma Oxt may diminish the expected effect of this peptide (decreased feeding and increased social functionality) and may account for hyperphagia and lack of satiety common in PWS. It has been suggested that a reduced number of Oxtr in PWS may lead to increased Oxt secretion by the posterior pituitary due to the loss of negative feedback. It is possible that the response to endogenous and exogenous Oxt differs [34]. This suggests the use of an Oxt antagonist to break down the negative feedback. In another study, the use of carbetocin, an Oxt analogue but with an improved receptor selectivity profile, was used in a randomized, placebo-controlled trial to improve the hyperphagia and behavioral symptoms of PWS patients aged 10–18. This study demonstrates significant improvement in these patients following 14 days of treatment, reducing compulsiveness and ameliorating overall functioning [35]. Liraglutide, a glucagon-like peptide-1 (GLP-1) receptor agonist that alters the activity of hypothalamic neurons in the arcuate nucleus directly associated with eating behavior, increasing satiety and reducing energy intake, thereby promoting weight loss, was also administered to PWS patients. However, Liraglutide did not result in a significant reduction in the BMI of children with PWS compared with placebo/no treatment but an apparent improvement in hyperphagia was seen [36]. Finally, the fourth clinical trial was conducted on the effect of Oxt on feeding and social behavior in infants of less than 6 months of life with PWS using unblended differing dosing schedules [37]. Escalating doses of Oxt over a 7-day period normalized suckling and improved swallowing regardless of Oxt doses. Striking long-term observational data implicated improvement in muscle tone and motor coordination as infants were able to crawl as toddler compared to untreated age-matched controls with PWS [4]. Indeed, 88% of subjects had normalization of suckling and improved swallowing immediately after treatment. Treated infants also had increased alertness, less fatigability and less social withdrawal. This improvement in gross motor skills was secondary to the improved muscle tone. As the treatment was given for only 1 week during infancy, this finding implies that a short course of Oxt at this age may have potential long-term benefits. The authors suggest that a positive feedback loop may explain these interesting results since exogenous Oxt promotes the continued secretion of endogenous hormones leading to long-term benefits. Moreover, the authors observed increased connectivity of the right superior orbitofrontal network after treatment with Oxt and suggest that this increased connectivity may also mediate long-term effects. Increased circulating acylated ghrelin, the active form of ghrelin, was monitored because of the effect of Oxt on ghrelin may also explain the normalization of feeding in this study. When giving IN-Oxt, it is important to consider timing and context (Table 2).

In conclusion, the best results with IN-Oxt are obtained in infants, toddler and sometimes in adolescents [37,38,39], albeit some positive effects were also reported in adults with PWS [40]. This suggests that there is probably a time-window during which Oxt system can be rewired and compensated due to neuroplasticity of the brain of infants [20]. Additionally, in infants the obesity is not established yet and muscles are not infiltrated with fat, as happen when the Oxt system is dysfunctional in adulthood, which makes it still possible to rescue the muscle phenotype of PWS individuals and consequently to normalize the Oxt-system [5,11]. In summary, the lack of consistency in the existing clinical trials shows that simply providing exogenous Oxt may not be sufficient to address the nature of the Oxt abnormality.

### 2.3. The Special Case of the Thermosensory and Pain Perception System in Prader–Willi Syndrome

Thermogenesis is the physiological response to thermoregulation, leading to heat production and retention under CS, while thermosensitivity to external temperatures begins at the skin level where different types of thermoreceptors are expressed allowing not only humans to differentiate between cold, warm and hot. Oxt regulates thermogenesis since Oxt/Oxtr signaling is a modulator of the thermogenic response to cold exposure by controlling BAT thermogenesis and energy expenditure by muscle contraction [11]. Oxt/Oxtr^−/−^ mice have impaired thermoregulation, resulting in thermosensory deficit and the incapacity to maintain a stable temperature. PWS and ASD individuals are hyposentive or hypersensitive to hot and cold and this seems to be related to the dysfunctional Oxt system that can lead to atypical sensory processing. Conversely, the atypical sensory understanding of the surrounding environment could lead to altered socio-cognitive behaviors [14]. Thermoregulation problems resulting in hypo or hyperthermia have been reported in children with PWS although detailed characteristics of thermoregulation problems and their clinical presentation remain unknown. Clinical cases indicate infants with PWS present two characteristics: hypotonic muscles and decreased sweating with extremely high body temperature similar to septic shock without any infections and caused by hypothalamic dysfunction [41]. Moreover, thermal deregulation and hyperpyrexia was found in a 13 years old boy with a confirmed genetic diagnosis of PWS and a diagnosis of thermal deregulation secondary to PWS [19]. In another case, accidental hypothermia was reported in an infant with PWS where a primary disturbance in parasympathetic autonomic regulation induced the drop in body temperature until 29.8 °C. Moreover, impaired pain and thermal stimulus perception with high pain threshold is a diagnostic criterion of PWS. These altered perceptions in PWS do not seem attributable to a peripheral nerve derangement due to metabolic factors or obesity but can be related to a reduction in sensory neurons at the level of dorsal root ganglia due to the absence of necdin expression [42]. Finally, in the Magel2 mouse model that lacks the PWS and Schaaf-Yang autism-associated gene, neonatal mice in a cold environment present hypo-reactivity while in control pups the pharmacogenetic inactivation of hypothalamic Oxt neurons mimicked this atypical thermosensory reaction found in Magel2 mutants, meaning that is caused by a deficit of the Oxt system [21]. This evidence supports the hypothesis that the thermoregulatory and pain sensitivity impairment in PWS are related to a dysfunctional Oxt system, and that Oxt’s regulation of thermogenesis may be the mechanistic explanation of this dysfunction in PWS as we will explain in the following chapter.

## 3. Oxytocin’s Regulation of Thermogenesis

### 3.1. Oxytocin’s Regulation of Thermogenesis in Genetic Studies

#### 3.1.1. Oxytocin in Bone, Brain and Brown Adipose Tissue after Cold Stress

Oxt/Oxtr^−/−^ mice show late-onset obesity but are normophagic for reduced metabolic rate [5]. For this reason, we speculated that the obesity caused by the lack of Oxt/Oxtr could be due to impaired thermoregulation and decreased core body temperature [6,43,44]. Following this rationale, we measured the mRNA levels of Oxt and Oxtr in the brain, bone, and BAT of mice after a short time 6 hour (6 h) and 5 days (5 d) CS. Oxt acts as a master gene in the brain since CS challenge upregulates Oxtr at 6 h and 5 d in the brain. The thermogenic challenge decreases Oxt in BAT after 6 h and increases Oxt in bone, which is consistent with the knowledge that Oxt modulates energy and bone [5,11,45]. After the short-term thermogenic challenge of 6 h, cold-stressed mice significantly increased their food intake, while no changes were observed in the abdominal fat pad and body weight compared to control mice. However, after a long-term cold exposure of 5 d the abdominal fat pad was significantly decreased while body weight returned to control values and food intake increased by threefold compared to control mice meaning that mice are in good health [7,11]. Oxt does not cross the blood–brain barrier [46]; however, Oxtr regulates the coordinated gene response to CS through a feed-forward loop in the brain [9], as shown by a regression study in which the elimination of Oxtr expression gene data in the brain causes the loss of gene expression at thermoneutrality but not after CS [10]. In the second part of our work, we studied the expression of Oxt in skeletal muscle, bone and brain, and we showed that the cross-talk between these organs allows the adaptation to cold stress, as we will describe in the following chapter [10,47].

#### 3.1.2. Oxytocin in Myosin after Cold Stress

Shivering thermogenesis is the involuntary contraction of skeletal muscles during cold exposure to generate heat. This process requires a great amount of energy and ATP [48] to sustain oxidative metabolism and lipolysis. CS potentiate slow fiber type [49], and Oxt regulates the adaptation of the organism to stressful situations [50] and thermoregulation since CS increases the c-Fos immunoreactivity of Oxt neurons in PVN [51]. However, the role of Oxt in skeletal muscles after cold exposure is unknown. This is why we speculated that Oxt might contract all slow-twitch muscle, as it does with the uterus, having a tonic, analgesic effect [52]. Specifically, in our experiments, we analyzed the mRNA expression level of Oxt/Oxtr in Soleus (Sol) and tibialis anterioris (TA) muscles following CS together with four myosin heavy chain (MyHC) isoforms, such as MyHC1, 2A, 2X and 2B, identified in skeletal muscles [53]. The different expressions of the four myosin heavy chain (MyHC) isoforms in skeletal phenotypes have been described previously [48,49,53,54,55,56]. Our data highlights the following four points: (1) In our experiments, the slow-twitch muscle phenotype is potentiated after CS [47]. Indeed, cold-stress down-regulates fast-twitch glycolytic myosin heavy-chain 2b (Mhc2b), while increasing the ratio of myosin heavy chain 1 (slow-oxidative)/myosin heavy chain 2b (fast-twitch glycolytic) expression in Sol but not in TA [10] consistent with the need of the slow-twitch oxidative muscle after thermogenic challenge and shivering thermogenesis. (2) Long-term CS for 5 d also downregulates Myhc2a in TA muscle but not Myhc1. The ratios Myhc1/Myhc2b and Myhc1/Myhc2a in Sol and TA muscles are increased after 6 h and 5 d CS, suggesting that thermogenic challenges affect the slow-twitch muscle and shifts TA muscle toward the slow-twitch phenotype while potentiating the slow-twitch phenotype of Sol. Myhc2x and Myhc1 isoforms are not affected in our experiments. (3) In addition, Oxtr is highly expressed in Sol after 5 d CS but not in TA. Androgen treatment up-regulates Oxt in skeletal muscles [57], and Oxt regenerates muscle from sarcopenia [58]. We hypothesized that Oxt exerts “tonic action”, increasing the tone of the slow-twitch muscle similarly to what occurred in the uterus [59]. (4) This Oxt circuit is mediated by the relation between brain and Sol through feed-forward/feed-back regulation, as shown by linear correlation analysis [10]. The brain Oxt may up-regulate the short-term response of Sol, while it may down-regulate the brain–Sol intercommunication after long-term exposure to CS [8,9,10]. This is a very important point and means that low circulating Oxt levels are required after long-term thermogenic challenges. However, the up-regulation of Oxtr in the brain and Sol is necessary to maintain the Oxt signaling after long-term CS, and this balances the decreased circulating Oxt consistent with previous studies [9] in what we called “The oxytonic effect”.

#### 3.1.3. Oxytocin and Transient-Receptor-Potential-Vanilloid-1 (TRPV1) and Pain Perception after Cold Stress

The Transient-Receptor-Potential-Vanilloid-1 (TRPV1) cation channel is a thermal and analgesic effector that interacts with Oxt/Oxtr mediating the Oxt analgesic effects [60,61]. Specifically, we explored the involvement of Oxtr/Triptophan vanilloid receptor 1 (TRPV1) genes and Oxt on the adaptation of skeletal muscles to CS in mice, and our results can be summarized in two important points: (1) CS induces the expression of TRPV1 and Oxtr in the skeletal muscles of Sol and TA and is higher in slow-twitch skeletal muscles [11]. Circulating Oxt leads to the activation of Oxtr and TRPV1 channel on membrane. (2) Oxtr and TRPV1 genes analyzed by regression analysis correlate in Sol and less in TA, and the correlation of Oxtr/TRPV1 in Sol and TA is present after CS but lost at thermoneutrality [11]. Oxt may lead to the activation of transmembrane ion channels permeable to calcium ions such as the TRPV1 cation channel [60,61]. We concludes that TRPV1 mediates the pain signaling of Oxt in neurons [47,60,61].

### 3.2. Oxytocin’s Regulation of Thermogenesis In Ex Vivo Studies

The genetic studies described above [11] were further confirmed by ex vivo studies, in which immunohistochemistry for Oxtr expression in PVN and SON and in the hippocampus (HIPP) and the measurement of circulating Oxt [11], were performed. These results can be summarized in the following four points: (1) Histomorphometric analysis shows that CS increases the expression of Oxtr in PVN and HIPP consistent with gene expression data in the whole brain [9]. (2) A different pattern of Oxtr expression is observed in SON, a major site of Oxt secretion, where Oxtr is unchanged after 6 h CS, but decrease at 5 d. (3) Circulating Oxt levels were unchanged after 6 h CS but significantly decreased after 5 d CS. (4) Oxt/Oxtr are expressed in cardiac muscle. For this reason, the heart of CS mice was also analysed. While the heart of control mice showed no signs of histopathology, the heart of mice exposed to 6 h CS showed histopathological signs such as necrosis and fibrosis that decreased in the heart of mice exposed to CS for 5 d when circulating Oxt decreased [11]. These results showed that in physiological condition Oxt augments the contractions of the myocardium and validate our theory in which Oxt increases the tonicity of skeletal muscles, including the heart. These were the first evidences of Oxt driven thermoregulation in cardiac muscle that were later confirmed by a study showing that Oxt released from PVN in the rostral medullary raphe region stimulates BAT thermogenesis and cardiac function triggering tachycardia and increasing metabolic rate [62]. In conclusion, the thermogenic challenge increases Oxtr in PVN and decreases circulating Oxt in a feed-back/feed-forward loop with the brain.

## 4. The Link with Prader–Willi Syndrome

**Figure 1:**
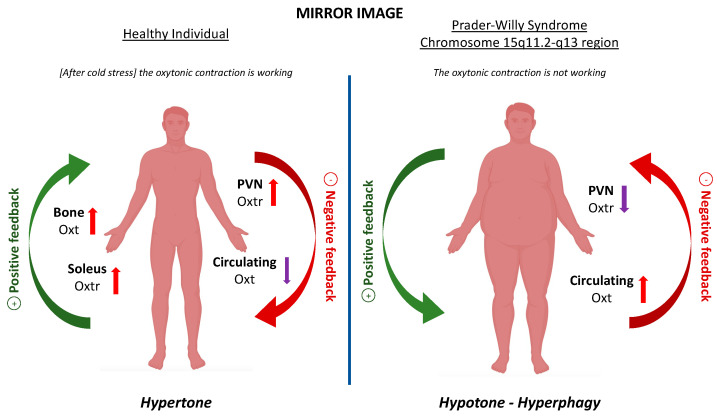
Schematic representation of Oxytocin physiologic mechanism in healthy individuals and dysfunctional mechanism in Prader–Willi Syndrome individuals.

The Oxt system in PWS appears dysfunctional. The main physiological features of PWS are reduced gene expression and density of Oxtr in PVN, as shown by post-mortem analysis [63], and increased circulating Oxt. Indeed, measurement of overnight fasting plasma Oxt levels in children with PWS compared to healthy control [29] showed that Oxt is higher in PWS children, and the genetic confirmation of PWS predicted the Oxt levels and vice versa [64]. The clinical characteristics of PWS such as hyperphagia and behavioural phenotype can be caused by the disrupted Oxt system between the brain and periphery described above. This phenotype of PWS individuals characterized by higher expression of Oxtr in PVN and low circulating Oxt is the striking mirror image of the phenotype of our model of cold-stressed mice in which CS turned on the Oxt system, increasing Oxtr mRNA in PVN and decreasing circulating Oxt [11]. The mirror image of our cold stress model is reduced Oxtr in PVN that leads to increased Oxt secretion by the posterior pituitary due to the loss of negative feedback like in PWS (Figure 1).

In our model of CS, where the Oxt system is functional, low temperature increased Oxtr in PVN and Sol and decreased circulating Oxt (Figure 1). This mechanism triggers the ‘oxytonic contractions’ that increases the tonicity of the slow-twitch muscle for shivering after CS. Conversely, the Oxt system is dysfunctional in Prader–Willi Syndrome individuals (PWS) that lack the expression of paternally active genes on chromosome 15q11.2-q13 region and are hypotonic and hyperphagic. In PWS, a reduced expression of Oxtr in PVN causes increased Oxt secretion by the posterior pituitary due to the loss of negative feedback. In healthy individual, the red arrow represents the negative feedback that decreases circulating Oxt following the increase in Oxtr in PVN. The green arrow represents the positive feedback that increases Oxtr in PVN following the decrease in circulating Oxt. In Prader–Willi Syndrome individuals, the red arrow represents the negative feedback that decreases Oxtr in PVN after the increase in circulating Oxt. The green arrow represents the positive feedback that increases circulating Oxt after the decrease in Oxtr in PVN.

PWS children have disrupted thermoregulation that can lead to hyperpyrexia and hypothermia with no detectable causes and that can be fatal [18,19,41]. This together with the sensory deficit that characterized PWS and ASD [14] adds further credence that Oxt’s regulation of thermogenesis is the mechanistic explanation of PWS. Moreover, PWS infants are hypotonic [65,66] while in our model of cold stress Oxtr mRNA was higher in slow-twitch muscle [11]. This is why we speculated that the dysfunctional Oxt system in PWS caused skeletal muscle hypotonicity in infants that drive late onset obesity at older age when muscle infiltrate with fat probably for the down regulation of Oxtr in this tissue and causes sarcopenic obesity. This hypothesis needs to be validated by an extensive study of Oxt/Oxtr in the skeletal muscles of PWS individuals. PWS clinical features suggests abnormalities of the hypothalamic–pituitary axis and hypothalamic syndrome. When PWS patients are treated with growth hormone, they improve with increased muscle size and decreased fat mass [67,68]. Circulating Oxt binds to Oxtr/TRPV1 in skeletal muscle, allowing adaptation to CS, having analgesic effect and increasing its tonicity [11]. However, overexposure of Oxtr to Oxt leads to the desensitization of Oxtr to protect cells from overstimulation, as reported for the majority of G protein-coupled receptors, including Oxtr [69]. This means that a longer therapeutic use of Oxt in PWS individuals can cause desensitization of Oxtr with no results. In this regard, a dose–response study with Oxt administration is advised [27,70,71]. In summary, a reduced expression of Oxtr in the PVN of PWS individuals, leads to increased Oxt secretion by the posterior pituitary due to the loss of negative feedback leading to loss of muscle tone and obesity. Conversely, in our model of cold stress, where the Oxt system is functional, low temperature increased Oxtr in the PVN and in Sol muscles, causing the decrease in circulating Oxt in plasma and the increased tonicity of the slow-twitch muscles [10,11]. Further studies will be necessary to measure Oxt/Oxtr in the skeletal muscles of PWS individuals to understand if dysfunctional “oxytonic effect” could be part of PWS.

## 5. Future Directions

The question arises if Prader–Willi Syndrome is an Oxytocin disease, albeit the lack of positive results from clinical trials suggest that simply providing exogenous Oxt may not compensate for the dysfunctional Oxt system. The high level of circulating Oxt detected in PWS individuals is in contrast with the appetite suppressant effect of Oxt and can be explained by the establishment of a sort of “Oxt resistance”, as seen with leptin [72]. At least three hypotheses have been proposed to explain why Oxt administration has not led to improvements. The first hypothesis regards the different forms of Oxt since Oxt is produced as a 12-amino-acid inactive form that goes through a cleaving process and is released as a 9-amino-acid active form [73]. PWS individuals have high circulating Oxt, which is Inconsistent with the anorexigenic effects of Oxt [29]. However, individuals with PWS may have too much of the inactive form of Oxt and not enough of the active form, which may be caused by a deficit of the cleaving process. This is confirmed by the decrease in prohormone convertase 1 (PC1) that is involved in cleaving Oxt in PWS-induced pluripotent stem cell-derived neurons. The second hypothesis is that Oxtr gene is suppressed in PWS because of a genetic defect or because of methylation of the gene since PWS patients present a reduction in Oxtr genes in PVN [13]. If these two hypotheses are confirmed, then treatment strategies may include manipulating either form of Oxt through pharmacological interventions or altering the epigenetic mechanism, such as methylation, to increase Oxtr expression. However, the fact that in infants a low dose of Oxt is as efficient as higher doses may be due to the stimulation of the endogenous somatodendritic release of Oxt via the Oxtr [37], indicating that in PWS infants, there may be a time-window when the administration of endogenous Oxt reset the dysfunctional Oxt system in brain and in skeletal muscles since infants are hypotonic but sarcopenic obesity is not yet established. This reports to the fact that Oxt potentiates the slow-twitch muscle, ameliorating hypotonicity, thus delaying the onset of sarcopenic obesity, as shown by the fact that PWS infants, after Oxt treatment, are able to crawl as toddlers. A third hypothesis, in light of the new results on thermogenesis, is that it may be worth administering an Oxt antagonist rather than Oxt/Oxt-agonist. This breaks down the negative feedback with PVN resetting “the oxytonic effect” and brings the Oxt system back to functionality, mimicking the functionality of the Oxt system activated after thermogenic challenge [11].

## 6. Conclusions

In summary, we need to develop a better understanding of Oxt abnormality in PWS also in light of the new discoveries on Oxt regulation of thermogenesis that will hopefully lead to more targeted and effective interventions. Of note are also the latest results on the effects of Oxt on muscle contraction that require better investigation in the specific muscle phenotypes of PWS individuals. Oxt’s regulation of thermogenesis and Oxt-driven muscle contraction are well related, especially in the manifestation of PWS. We hope that our study will soon translate into clinical research to understand to which extent Oxt is involved in PWS.

## Figures and Tables

**Table 1:** Features of Prader–Willi Syndrome patients.

Nutritional Phase 1a: infants	Severe neonatal hypotonia with poor appetite and problems sucking necessitating assisted feeding.
Nutritional Phase 1b: around age 2 years	Improved appetite.
Nutritional Phase 2°: age 2 to 3 years	Weight gain without a change in appetite.
Nutritional Phase 2b: age 4–5 years	Increased interest in food with increased anxiety and behavioral problems.
Nutritional Phase 3: age 8	Insatiable appetite with worsening behavioral problems combined with other endocrine problems due to hypothalamic dysfunction. These include episodes of hyperpyrexia with no infection and/or hypothermia with no causes, abnormal detection of cold and hot, abnormal perception of pain, cognitive and growth delay and autistic spectrum disorder. Post-mortem analysis indicated a decrease in Oxytocin receptor level in brain of Prader–Willi Syndrome patients and an increase in plasmatic Oxytocin.

**Table 2:** Main clinical intervention using Oxytocin in Prader–Willi Syndrome patients.

First study [32]	24 children aged 5–11 years with or without PWS diagnosis. 5 days IN-Oxt or placebo/4 weeks washout and again 5 days IN-Oxt or placebo. Positive but not significative trends of improvement in behavioral and appetite parameters.
Second study [33]	26 children aged 3–11 years. 8 weeks placebo double blind IN-Oxt randomized trial. Positive trends were found in boys but not in girls, and patients with a deletion had a significant positive effect but not patients with maternal uniparental disomy associated with more severe symptoms.
Third study [34]	Children aged 5–18 years. Placebo was associated with improvement in hyperphagia and behavior but not the treatment with Oxytocin.
Fourth study [37]	18 Infants under 6 months old. Escalating doses of Oxytocin administered over a 7 day period. Normalization of suckling, improved swallowing, improvement in muscle tone and motor coordination. Improvement in gross motor skills also in long-term effects since treated patients were able to crawl as toddlers.

## Data Availability

Not applicable.
